# Serum concentrations of PFASs and exposure-related behaviors in African American and non-Hispanic white women

**DOI:** 10.1038/s41370-018-0109-y

**Published:** 2019-01-08

**Authors:** Katherine E. Boronow, Julia Green Brody, Laurel A. Schaider, Graham F. Peaslee, Laurie Havas, Barbara A. Cohn

**Affiliations:** 1 Silent Spring Institute, Newton, MA USA; 2 University of Notre Dame, Notre Dame, IN USA; 3 Child Health and Development Studies Participant Advisory Council, Berkeley, CA USA; 4 Child Health and Development Studies, Public Health Institute, Berkeley, CA USA

**Keywords:** PFAS, Personal exposure, Dental floss, Food Packaging, Drinking water

## Abstract

Per- and polyfluoroalkyl substances (PFASs) are used in a wide range of consumer products for their water- and grease-resistant properties, but few studies have explored this exposure route. We used multiple regression to investigate associations between six self-reported behaviors hypothesized to influence PFAS exposure and serum concentrations of six PFAS chemicals in 178 middle-aged women enrolled in the Child Health and Development Studies, about half of whom are African American. Blood samples were collected in 2010–2013, and participants were interviewed about behavior in 2015–2016. Results showed that African American women had lower levels of perfluorooctanoic acid (PFOA) and perfluorohexanesulfonic acid (PFHxS) compared with non-Hispanic white women. In African Americans, but not others, frequent consumption of prepared food in coated cardboard containers was associated with higher levels of four PFASs. Flossing with Oral-B Glide, having stain-resistant carpet or furniture, and living in a city served by a PFAS-contaminated water supply were also associated with higher levels of some PFASs. Product testing using particle-induced γ-ray emission (PIGE) spectroscopy confirmed that Oral-B Glide and competitor flosses contained detectable fluorine. Despite the delay between blood collection and interview, these results strengthen the evidence for exposure to PFASs from food packaging and implicate exposure from polytetrafluoroethylene (PTFE)-based dental floss for the first time.

## Introduction

Per- and polyfluoroalkyl substances (PFASs) are a commercially important group of chemicals with wide applications because of their unique ability to resist both water and lipids. In addition to specialized industrial applications and use in fire-fighting foams, PFASs are frequently used in consumer products. Most commonly, they are used in nonstick and water-, stain-, or grease-resistant coatings, which are applied to a diverse range of products, including food packaging, cookware, carpet, furniture, textiles, and outdoor performance gear. Given their extensive use and persistent nature, it is unsurprising that PFASs have been detected in water and soil [1], and in the bodies of almost all Americans [2]. Exposure to the long-chain PFASs perfluorooctanoic acid (PFOA) and perfluorooctanesulfonic acid (PFOS) has been linked to kidney and testicular cancer, decreased semen quality, and ulcerative colitis in adults [3–5], and to thyroid disease, immune response, and lowered sex and growth hormones in children [6–8].

Serum PFAS levels in the general population differ by race. Analyses of data from multiple cycles of the National Health and Nutrition Examination Survey (NHANES) show consistent differences in PFAS exposures by race. Differences between non-Hispanic whites and blacks vary by chemical, with whites having higher levels of PFOA and blacks having higher levels of perfluorononanoic acid (PFNA) and, to a lesser extent, PFOS [9–12]. These differences persist after controlling for family income [12]. In Project Viva—a study of children aged 6–10 born in the Boston area—children of black mothers had lower levels of PFOA, PFOS, perfluorohexanesulfonic acid (PFHxS), and 2-(N-methyl-perfluorooctane sulfonamido) acetic acid (Me-PFOSA-AcOH), but not PFNA, compared with children of white mothers, even after adjusting for maternal concentration during pregnancy [13]. The factors contributing to these observed differences by race are not well understood, but could result from differences in exposure-related behaviors and community-level exposures.

Environmental contamination, including exposure mediated by diet, is a large contributor to individual PFAS levels, even in communities not directly impacted by industrial operations. In a study of California women, participants whose drinking water supplies had detectable concentrations of PFOA and PFOS had median serum levels of the chemicals elevated by 38 and 29%, respectively, compared with participants whose drinking water did not have detectable concentrations [14]. Fish and shellfish are widely contaminated by PFASs [15, 16], and seafood consumption is associated with PFAS levels [17, 18]. Other unprocessed foods (e.g., vegetables, meat, eggs) typically contain lower levels of PFASs than fish and shellfish [19, 20].

PFASs and their precursors are found in a wide array of consumer products. They are used in food packaging and contact materials [21–25] and can migrate into food during typical use and preparation [22, 26–28]. PFASs are also present in indoor environments [29, 30], where they are released to air and dust from consumer products. Fabrics including apparel and uniforms, home and outdoor textiles, carpet, and upholstered furniture are often treated with PFASs prior to sale, and many cleaning and treatment products contain PFASs as well [25, 31, 32]. Other consumer products known to contain PFASs based on product testing include dental floss, nonstick cookware, ski and floor waxes, and thread seal tape [25, 31, 32].

Given the numerous sources of PFASs in everyday diets and environments, it is difficult to pinpoint which behaviors contribute most significantly to PFAS exposure. Many studies have focused exclusively on dietary sources [11, 18, 20, 28, 33, 34]. Two studies to date have attempted to link both dietary and non-dietary sources to measured exposure levels [13, 35]. Harris et al., in their analysis of 545 children in Project Viva, found positive associations between sleeping (but not awake time) in a carpeted room and levels of PFOS, PFHxS, and Me-PFOSA-AcOH, mixed associations with time spent outdoors and PFAS levels depending on the season, and suggestive positive associations with high fast-food consumption and PFOA, PFNA, and Me-PFOSA-AcOH, after adjusting for child and maternal characteristics [13]. In their analysis of 68 children and 149 adults living in the Central Valley of California, Wu et al. found, in addition to dietary predictors, positive associations in adults between occupational exposure or having used fire extinguisher and levels of PFOA, PFNA, PFHxS, and PFOS, and positive associations in children between wearing waterproof clothing and PFOS and PFNA [35]. No associations were found with housing characteristics or use of nonstick cookware, stain-repellent products, or polishing and coating products. These studies provide preliminary insights on product and behavioral contributions to PFAS exposure, but additional data are needed to evaluate differences by race and contributions from previously unstudied behaviors.

In this paper, we examine the role of self-reported consumer product use in predicting exposure levels of six PFAS chemicals in a cohort of middle-aged women, half of whom are African American. We include several exposures in common with previous analyses (e.g., consumption of microwave popcorn) and for the first time consider exposure from Oral-B Glide dental floss. We also present data on the presence of fluorine in Oral-B Glide and other dental flosses as an indicator of polytetrafluoroethylene (PTFE) in these products. This study extends work on consumer product exposure to PFAS and evaluates whether consumer behaviors mediate differences in PFAS exposure by race.

## Methods

### Study population

**Fig. 1:**
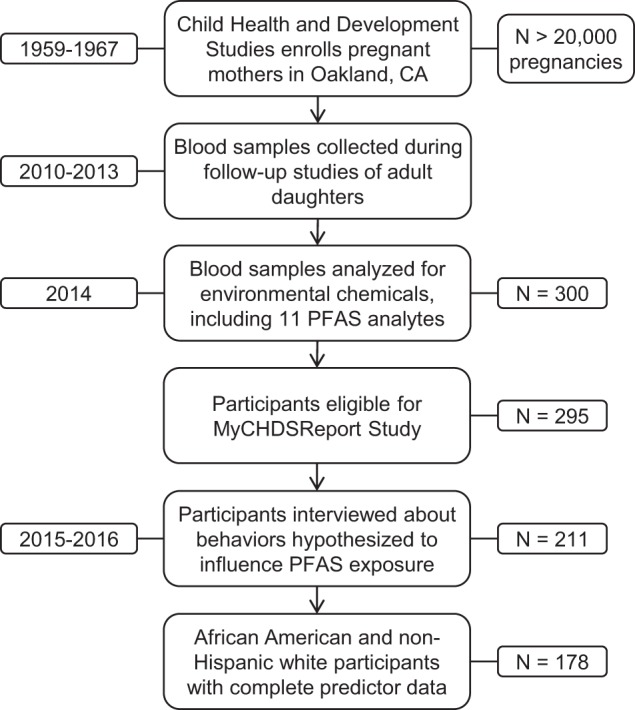
Overview of the CHDS study population and data used in all analyses

Participants were drawn from the Child Health and Development Studies (CHDS), a multigenerational cohort study that enrolled pregnant mothers in Oakland, CA from 1959 to 1967 [36]. CHDS collected blood samples from second-generation daughters in September 2010 to March 2013 and tested 300 women (150 African American and 150 non-African American) for environmental chemicals as a part of the Three Generations Study. Of these women, 295 were eligible to participate in the MyCHDSReport Study, which conducted interviews to examine the effect of returning biomonitoring results on participants’ exposure-related knowledge, behavior, and attitudes [37]. The MyCHDSReport Study and earlier blood sampling were approved by the Institutional Review Board at the Public Health Institute, and informed consent was obtained from all participants. Figure 1 provides an overview of the study population and key phases of data collection.

### PFAS measurements

Blood samples were analyzed in 2014 by the Environmental Chemistry Lab at the California Department of Toxic Substances Control for 42 chemicals, including 11 PFAS analytes: perfluoroheptanoic acid (PFHpA), PFOA, PFNA, perfluorodecanoic acid (PFDeA), perfluoroundecanoic acid (PFUnDA), perfluorododecanoic acid (PFDoDA), PFHxS, PFOS, perfluorooctanesulfonamide (PFOSA), 2-(N-ethyl-perfluorooctane sulfonamido) acetic acid (Et-PFOSA-AcOH), and Me-PFOSA-AcOH. PFAS analytes were measured using the online SPE-HPLC-MS/MS method and quality control procedures as described previously [38]. Detection limits for the analytes are reported in Table 1.

**Table 1:** Range and percentiles of 11 PFAS serum concentrations measured in middle-aged women (*n* = 178) in the Child Health and Development Studies from 2010 to 2013. Analytes are ordered by descending detection frequency and median concentration

PFAS analyte (ng/mL)	MDL	% Detect	Min	Percentile			Max
				10th	50th	90th	
PFOS	0.2	100	0.34	1.85	4.74	10.4	21.5
PFOA	0.064	100	0.13	0.66	1.8	3.44	12.1
PFHxS	0.018	100	0.07	0.35	0.97	2.48	16.5
PFNA	0.019	100	0.14	0.39	0.79	1.29	8.25
PFDeA	0.02	100	0.02	0.1	0.22	0.49	1.85
Me-PFOSA-AcOH	0.039	99	nd	0.08	0.18	0.7	2.8
PFOSA	0.019	78	nd	nd	0.03	0.13	0.4
PFHpA	0.0014	76	nd	nd	0.02	0.08	0.44
PFUnDA	0.092	70	nd	nd	0.14	0.31	0.79
Et-PFOSA-AcOH	0.041	32	nd	nd	nd	0.09	0.65
PFDoDA	0.24	2	nd	nd	nd	nd	1.02

For comparison, we examined PFAS measurements collected in the 2011–2012 cycle of NHANES. We did not include data from the 2009–2010 or 2013–2014 NHANES cycles because our sample collection period overlapped with these 24 month cycles by only 4 and 3 months, respectively. For relevance to our study population, we limited the NHANES comparison group to women aged 40–60 years who were born in the US and of either non-Hispanic black or non-Hispanic white race/ethnicity.

### Demographic and behavioral variables

Self-reported race and highest level of education were obtained by interview at the time of blood collection. Highest level of education was categorized into less than a Bachelor’s degree (high school or less, Associate’s degree, technical or vocational training) or at least a Bachelor’s degree (Bachelor’s, Master’s, doctoral, or professional degree). Self-reported race/ethnicity was categorized as African American if the participant indicated African American as one of her races to a question allowing multiple response categories for race and ethnicity. Participants were categorized as non-Hispanic white if they indicated only “White, Caucasian, European, not Hispanic.” We excluded other race/ethnicities from analysis to establish homogeneous subgroups.

Data on behaviors hypothesized to influence PFAS exposure were collected between July 2015 and April 2016 as a part of the MyCHDSReport Study. Women were interviewed before and after receiving a report showing individual or aggregate biomonitoring results for several classes of environmental chemicals along with contextual information about the chemicals and overall findings from the study [39]. The behavioral data presented here are from the pre-interview (before participants received their results report). The Survey Research Group (SRG) at the Public Health Institute interviewed 165 women with a structured questionnaire, and Silent Spring Institute conducted semi-structured interviews with 46 women. Participants were randomly assigned to interview type. This mixed methods approach allowed us to elicit rich qualitative detail from the semi-structured interviews while also gathering quantitative data. Both interview groups were asked identical sets of closed-ended, Likert-type questions about behaviors expected to be related to PFAS exposure. In the semi-structured group, in addition to the categorical responses, we have a record of any other comments made by the participant, which sometimes included asking clarifying questions or providing additional contextual information. Women were asked about nine behaviors related to potential PFAS exposure, including six questions about food consumption, one on dental flossing, and two on stain-resistant treatments applied to furniture and carpets (see Table S1 for questions and response levels). Seafood consumption was asked only of the subset of women interviewed by SRG.

We evaluated the possible contribution of public drinking water contamination using data reported under the U.S. Environmental Protection Agency’s third Unregulated Contaminant Monitoring Rule (UCMR3) [40]. UCMR3 required assessment monitoring in all public water supplies serving more than 10,000 people and in 800 representative public water supplies serving fewer than 10,000 people between 2013 and 2015. We identified public water supplies with a detectable level of any of six measured PFASs (PFHpA, PFOA, PFNA, PFBS, PFHxS, and PFOS) in the UCMR3 data in states where participants lived at the time of their blood draw. To determine the cities served by each water supply, we used information from the Safe Drinking Water Information System (SDWIS) [41] or a description of the service area from the water supply’s website when SDWIS data were incomplete. We compared the list of cities served by PFAS-contaminated supplies against participants’ addresses at the time of their blood draws and recorded positive matches.

### Statistical analysis

All analyses were restricted to participants categorized as African American or non-Hispanic white with complete data for the demographic and behavioral variables (*n* = 178). Incomplete data resulted when participants declined to answer or answered “don’t know” for any question. We used descriptive statistics to explore PFAS concentrations in the study population and in comparison with data from the 2011 to 2012 cycle of NHANES. Chemicals detected in ≤ 80% of participants (Et-PFOSA-AcOH, PFDoDA, PFHpA, PFOSA, PFUnDA) were excluded from further analysis to avoid the challenges associated with left-censored data. The remaining chemicals (PFDeA, PFHxS, PFNA, PFOA, PFOS, Me-PFOSA-AcOH) were detected in > 98% of participants; we substituted the limit of detection divided by two for three participants who did not have detectable levels of Me-PFOSA-AcOH. Concentration data were natural-log transformed to meet assumptions of normality. We examined differences in natural-log transformed PFAS concentrations by race using Welch’s two sample *t* test in CHDS and a survey-based two sample *t* test equivalent in NHANES (with the exception of Me-PFOSA-AcOH in NHANES only, for which we used a survey-based Wilcoxon rank-sum test equivalent and untransformed concentrations to account for its lower detection frequency of 60%). We calculated Spearman rank correlations to assess relationships among PFASs.

Based on the distributions of the variables, collinearity, and current understanding of exposure sources, we combined behaviors and response levels as described in Table 2. Eating pizza, French fries, and other takeout from coated cardboard containers were summed into a single predictor about eating prepared food from coated cardboard containers and categorized into “never,” “low,” and “high.” Applying stain-resistant treatment and acquiring already-treated furniture and carpet were summed into a single predictor and categorized into “none” and “one or more.” Flossing with Oral-B Glide, eating food prepared with nonstick cookware, and eating microwave popcorn were converted into binary variables representing “never” or “ever.” We used two-sided Fisher’s exact tests to examine differences in frequency of behaviors by race.

**Table 2:** Description of the behaviors used in regression analysis and hypothesized to influence PFAS exposure

Behavior	Description	Response levels
	During the past month…	
Non-stick cookware	How often did you eat food prepared using non-stick cookware?	Never, ever
Microwave popcorn	How often did you eat popcorn made in microwave popcorn bags?	Never, ever
Glide floss	How often did you use Oral-B Glide dental floss?	Never, ever
Coated cardboard containers	How often did you eat pizza, french fries, or other takeout food from coated cardboard containers?	Never, low, high^a^
Seafood	Did you eat any fish or seafood purchased at the grocery store or caught in California waters?	Never, ever
	In the past five years…	
Stain-resistant carpet and furniture	How many pieces of furniture or carpets in your home are treated for stain-resistance (pre-treated or spray treatment applied in-home)?	None, one or more

^a^Response levels for the individual variables (never or almost never, several times a month, two or more times a week, and every day) were converted to day-equivalents (1, 4, 12, and 28 days) and summed. Response levels for the combined variable are defined as ≤ 3 days (never), 6–14 days (low), and ≥ 17 days (high)

We first used linear models to examine the interaction between race and each predictor independently on natural-log transformed concentration of each PFAS. Next, we used multiple regression to examine mutually adjusted associations between predictor variables and natural log-transformed PFAS concentration. Models included race, education, food from coated cardboard containers, food prepared with nonstick cookware, microwave popcorn, stain-resistant furniture and carpets, flossing with Oral-B Glide, and living in a city served by a PFAS-contaminated water supply as predictor variables. We included race by predictor interaction terms in the mutually adjusted model when the interaction was significant (α = 0.1) in the unadjusted linear model. In the mutually adjusted model, we assessed significance of all terms at α = 0.05. Because PFASs bind primarily to proteins in the serum and liver [42], we do not expect circulating levels of PFASs to be influenced by an individual’s body fat; thus, we did not include body mass index in our analyses. We included seafood consumption in a separate analysis limited to the SRG interviews. We back-transformed coefficient estimates and confidence intervals using the equation (*e*^*β*^−1)*100 to estimate the percent change in PFAS concentration associated with each predictor.

Statistical analysis was completed using R version 3.5.0 [43]. NHANES data were accessed and analyzed using appropriate weighting techniques with the RNHANES [44] and survey [45] packages. R code will be made available upon reasonable request.

### Dental floss testing

Certain dental flosses, specifically including Oral-B Glide, have been reported to be manufactured from PTFE [46, 47]. To evaluate the plausibility of PFAS exposure from Oral-B Glide and other brands, we screened 18 floss products for the presence of fluorine as an indicator of PTFE. This analysis provides an initial evaluation of the availability of dental flosses that may contain PFAS compounds. Dental floss samples were analyzed for total fluorine using particle-induced γ-ray emission (PIGE) spectroscopy at the Hope College Ion Beam Analysis Laboratory. Methods were adapted from those described in Ritter et al. [48], and details are included in the Supplementary information. PIGE has previously been applied to infer PFAS content of other solid materials [21, 49]. Samples from 26 packages of dental floss representing 18 products were tested, blind to brand name (Table S4). Inorganic fluorine standards, blanks, and duplicate samples for five packages of floss were analyzed as part of quality control protocols. Products were collected on an ad hoc basis to represent a range of brands and floss types and to include products readily available to consumers within the past decade. This was intended as a rapid, low-cost chemical analysis to aid in the interpretation of the self-reported data about flossing.

## Results

### Characteristics of the study population

Of 295 eligible women, 211 (72%) completed the first interview of the MyCHDSReport Study. One hundred and seventy-eight (84%) had complete predictor data and identified as either African American (*n* = 87) or non-Hispanic white (*n* = 91), meeting inclusion criteria for this analysis. Participants excluded from this analysis included 18 participants (9%) of other race/ethnicity and having complete data (nine Hispanic or Latino, four Asian, and five mixed race with no African American identity) and 15 participants (7%) lacking complete data.

Participants were between 48 and 56 years old at the time of the interviews. Ninety-four percent lived in California and the remaining participants were located across the United States. More non-Hispanic whites had at least a Bachelor’s degree than African Americans (53% vs. 38%, Table 3).

**Table 3:** Frequencies by race of participant behaviors and characteristics hypothesized to predict PFAS exposure and results of two-sided Fisher’s exact tests for differences in frequency by race

	*N* (%)			*N* (%)
Predictor response levels	Non-Hispanic white	African American	*p*	All
*Seafood*				
Never	23 (34)	21 (30)	0.72	44 (32)
Ever	45 (66)	48 (70)		93 (68)
Not asked	23	18		41
*Glide floss*				
Never	46 (51)	48 (55)	0.55	94 (53)
Ever	45 (49)	39 (45)		84 (47)
*Non-stick cookware*				
Never	20 (22)	21 (24)	0.86	41 (23)
Ever	71 (78)	66 (76)		137 (77)
*Coated cardboard containers*				
Never	32 (35)	27 (31)	0.21	59 (33)
Low	53 (58)	47 (54)		100 (56)
High	6 (7)	13 (15)		19 (11)
*Microwave popcorn*				
Never	74 (81)	63 (72)	0.21	137 (77)
Ever	17 (19)	24 (28)		41 (23)
*Stain-resistant carpet and furniture*				
None	49 (54)	57 (66)	0.13	106 (60)
One or more	42 (46)	30 (34)		72 (40)
*Education*				
Less than bachelor’s	43 (47)	54 (62)	0.052	97 (54)
Bachelor’s or more	48 (53)	33 (38)		81 (46)
*City served by a PFAS-contaminated water supply*				
No	89 (98)	83 (95)	0.44	172 (97)
Yes	2 (2)	4 (5)		6 (3)

Data were collected from middle-aged women (*n* = 178) in the Child Health and Development Studies from 2015 to 2016

### Overall PFAS concentrations

**Fig. 2:**
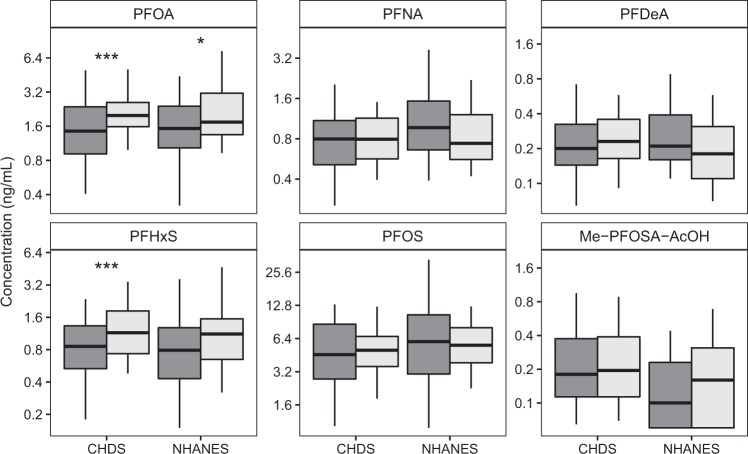
The distribution of serum PFAS concentrations for participants in CHDS compared with similar women from the National Health and Nutrition Examination Survey (NHANES). The whiskers indicate the 5th and 95th percentiles, and the boxes show the 25th, 50th, and 75th percentiles. Data are plotted on log scale. Dark gray bars are African Americans and light gray bars are non-Hispanic whites. NHANES data are from the 2011 to 2012 cycle and subset to 40–60-year-old women born in the United States of non-Hispanic black or non-Hispanic white race/ethnicity. Asterisks indicate a significant difference by race within CHDS or within NHANES (*p < 0.05; ***p < 0.001)

Of 11 PFASs measured in this study, six were retained for analysis. PFOS was detected at the highest concentrations, with a median of 4.74 ng/mL (interquartile range [IQR] = 3.04–8.10), followed by PFOA, with a median of 1.80 ng/mL (IQR = 1.21–2.47). Table 1 presents descriptive statistics. PFOA, PFNA, PFDeA, and PFOS were strongly and significantly correlated with each other with Spearman correlation coefficients ranging between 0.65 and 0.73, while Me-PFOSA-AcOH was weakly correlated with all other analytes (0.08–0.21) (Table S2). Levels of PFHxS and PFOA differed by race, with non-Hispanic whites having a 34% higher median level of PFHxS and a 37% higher median level of PFOA than African Americans (Fig. 2). Higher levels of PFOA in non-Hispanic whites were also observed in NHANES (Fig. 2).

Participants in this study had PFAS exposures largely comparable with similar-aged women in NHANES, with the exception of Me-PFOSA-AcOH (Fig. 2). The median level of Me-PFOSA-AcOH in African American participants was 80% higher than in non-Hispanic black women in NHANES, but similar to levels in non-Hispanic white women in our study and NHANES.

### Predictors of PFAS exposure

Among behaviors we thought might predict PFAS levels, eating food prepared with nonstick cookware was the most frequently performed (77% of participants do this at least several times a month), while consuming microwave popcorn was the least frequent (23% of participants do this at least several times a month). Frequencies of self-reported behaviors are presented in Table 3 (see Table S1 for frequencies by original questions and response levels before they were combined into summary variables). Frequencies of PFAS-related behaviors did not differ by race (Table 3). Six participants (3.4%) lived in cities served by a PFAS-contaminated water supply, consistent with a national estimate that 4% of public water supplies have detectable levels of PFAS [50].

Regression analysis occurred in two phases. Examining the interaction between race and each behavior with each log-transformed PFAS concentration, we observed that race modified the relationship between frequently consuming prepared food in cardboard containers and levels of PFOA, PFNA, PFDeA, and PFOS; the relationship between having stain-resistant carpet or furniture and PFDeA; and the relationship between education and PFHxS (Table S3). These interaction terms were therefore included in the mutually adjusted regression models.

In the mutually adjusted models, we found that African American race was associated with 52.6% (95% CI: 34.4–65.8) lower levels of PFOA (Table 4). Flossing with Oral-B Glide was associated with 24.9% (95% CI: 0.2–55.7) higher levels of PFHxS. Living in a city served by a PFAS-contaminated water supply was associated with higher levels of PFOA (100.3%, 95% CI: 18.2–239.5), PFNA (83.6%, 95% CI: 16.6–189.2), and PFHxS (103.5%, 95% CI: 10.3–275.2), although the small number of participants living in PFAS-contaminated water districts (*n* = 6) contributed to large variances associated with these estimates. Having stain-resistant carpet or furniture was associated with higher levels of PFNA (18.7%, 95% CI: 0.5–40.2) in all participants and PFDeA (39.6%, 95% CI: 5.9–84.2) in non-Hispanic whites only.

**Table 4:** Mutually adjusted associations between participant behavior and serum concentration of six PFASs (*n* = 178) with tests for statistical interaction by race for predictors with significant interactions in the unadjusted models

Predictor	Percent change in PFAS concentration (95 percent confidence interval)					
Response levels	PFOA	PFNA	PFDeA	PFHxS	PFOS	Me-PFOSA-AcOH
Intercept	95.8 (43, 168)	−26.7 (−44.1, −3.9)	−78.4 (−84.7, −69.6)	−1.3 (−30.1, 39.5)	307.1 (187, 477.5)	−84.1 (−89.5, −76)
*Glide floss*						
Never	ref	ref	ref	ref	ref	ref
Ever	4.8 (−13.4, 26.7)	7 (−9.2, 26.1)	1 (−17.5, 23.5)	24.9 (0.2, 55.7)*	8.7 (−12, 34.4)	12.7 (−14.2, 48)
*Non-stick cookware*						
Never	ref	ref	ref	ref	ref	ref
Ever	4.3 (−16.7, 30.6)	−2.7 (−19.9, 18.1)	−18 (−35.4, 4)	−14.2 (−33.9, 11.4)	−11 (−30.7, 14.3)	5.1 (−23.8, 45)
*Microwave popcorn*						
Never	ref	ref	ref	ref	ref	ref
Ever	18.7 (−5.7, 49.4)	8.8 (−10.8, 32.6)	24 (−2.8, 58.2)	6.1 (−18.7, 38.6)	26.3 (−2.3, 63.1)	−12.4 (−37, 22)
*City served by a PFAS-contaminated water supply*						
No	ref	ref	ref	ref	ref	ref
Yes	100.3 (18.2, 239.5)*	83.6 (16.6, 189.2)**	67.9 (−4, 193.6)	103.5 (10.3, 275.2)*	62.8 (−9.5, 192.7)	52.4 (−28.3, 224)
*Race*						
Non-Hispanic white	ref	ref	ref	ref	ref	ref
African American	−52.6 (−65.8, −34.4)***	−21.4 (−40.6, 4)	−21.6 (−46.7, 15.4)	−18.9 (−39.5, 8.9)	−26.1 (−48.6, 6.2)	3.2 (−21.4, 35.6)
*Education (Bachelor’s degree or more)*						
No	ref	ref	ref	ref	ref	ref
Yes*Race	–	–	–	*	–	–
Yes (NHW)	−7.5 (−24, 12.5)	−11.2 (−25, 5.1)	12.9 (−8.2, 39)	40.4 (3.5, 90.6)*	11.7 (−10.2, 38.9)	7 (−19.3, 41.7)
Yes (AA)				−13.8 (−37.8, 19.5)		
*Stain-resistant carpet and furniture*						
None	ref	ref	ref	ref	ref	ref
One or more*Race	–	–	NS	–	–	–
≥One (NHW)	2.8 (−15.3, 24.7)	18.7 (0.5, 40.2)*	39.6 (5.9, 84.2)*	−12.3 (−29.8, 9.7)	2.5 (−17.4, 27.1)	5.8 (−19.7, 39.5)
≥One (AA)			2.1 (−24.4, 37.8)			
*Coated cardboard containers*						
Never	ref	ref	ref	ref	ref	ref
Low*Race	NS	NS	NS	–	NS	–
Low (NHW)	2.9 (−22.1, 35.8)	7.7 (−15.2, 36.8)	−1.6 (−26.7, 32)	7.9 (−14.6, 36.4)	21.9 (−10.5, 66.1)	22.1 (−8.7, 63.2)
Low (AA)	45.4 (7.8, 96)*	18.4 (−8.5, 53.1)	26.1 (−8.2, 73.2)		38.3 (−0.8, 92.8)	
High*Race	**	**	**	–	*	–
High (NHW)	−36.9 (−63.8, 10)	−12.4 (−45.7, 41.3)	−26.7 (−59.3, 32.1)	10 (−25.4, 62.3)	−23.2 (−58.6, 42.6)	40.7 (−13, 127.6)
High (AA)	99.9 (31.1, 204.8)**	95.9 (36.3, 181.7)***	124.4 (43.2, 251.5)***		89.6 (18.6, 203.1)**	
Model R^2^	0.21	0.16	0.16	0.17	0.12	0.04

NS, *p* > 0.05; *p ≤ 0.05; **p ≤ 0.01; ***p ≤ 0.001

For tests of interaction, we report the overall significance of the interaction term and the magnitude and significance of the race-specific marginal associations. Interactions not included in a model are indicated by dash and the non-conditional marginal association is reported

**Fig. 3:**
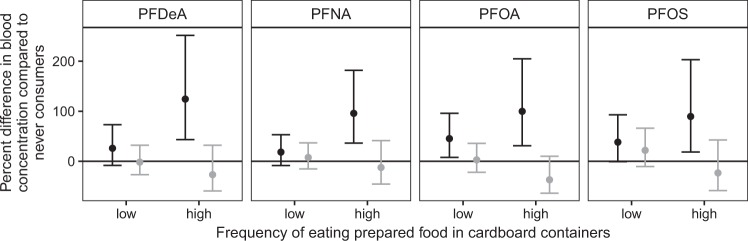
Race-specific marginal effect estimates for percent change in PFAS exposure from consuming prepared food in cardboard packaging. Dark gray symbols are African Americans and light gray symbols are non-Hispanic whites

In addition, we found significant statistical interactions on the multiplicative scale between eating high levels of prepared food in cardboard packaging and race and levels of PFOA, PFNA, PFDeA, and PFOS. The conditional marginal effects of eating “high” levels of prepared food in cardboard packaging on PFAS level in African Americans ranged from 89.6 to 124.4%, and “low” consumption of prepared food was also associated with a 45.4% increase in PFOA compared with “never” (Table 4, Fig. 3). The conditional marginal effects of eating prepared food in cardboard packaging on PFAS level did not show elevated levels in non-Hispanic whites. Only 19 participants (of any race) consumed high levels of prepared food in cardboard containers, contributing in part to the large variance associated with these estimates. There was also a statistically significant race by education interaction for PFHxS level. In non-Hispanic whites, having at least a Bachelor’s degree was associated with 40.4% (95 CI: 3.5–90.6) higher levels of PFHxS; the association was not significant in African Americans. Seafood consumption was not associated with PFAS levels in the analysis restricted to the subset of participants (*n* = 137) queried about this aspect of diet (data not shown).

To better understand the interaction between eating high levels of prepared food in cardboard packaging and race, we looked separately at the three behaviors that comprise the prepared food variable. Results showed a significant difference between African Americans and non-Hispanic whites in the frequency of consuming French fries (but not pizza or other takeout in cardboard containers), with 49% of African Americans reporting eating French fries at least several times a month compared with 19% of non-Hispanic whites (two-sided Fisher’s exact test, French fries: *p* < 0.001; pizza: *p* = 0.31; takeout: *p* = 0.14).

### Fluorine in dental floss

Fluorine was detected in six out of 18 dental floss products, including all three products branded as Glide floss and two of three products with label statements inviting consumers to “compare to Oral-B Glide” floss (Table S4). PIGE analysis yielded typical values between 1000 and 3000 counts/microCoulomb of total fluorine for these six floss samples. It was not possible to calculate absolute quantities of fluorine in these samples because the floss was typically narrower than the ion beam and the floss shape varied between samples. In comparison, the limit of detection was around 100 counts/microCoulomb of fluorine (based on inorganic fluorine standards), and broad polymeric PTFE tape intersecting the entire beam yielded values around 10,000 counts/microCoulomb. Floss samples having no detectable fluorine signature were considered non-fluorinated and are thus unlikely to contain PTFE. All duplicate pairs (*n* = 5) from the same floss package were in agreement, as were all samples from multiple packages of the same product (Table S4).

## Discussion

This study strengthens the evidence that PFAS exposure is influenced by product use and varies by race. In our cohort of middle-aged women, non-Hispanic white women had higher blood levels of PFOA and PFHxS compared with African Americans. These findings are consistent with previous reports of differences by race [9, 12, 13]. Consumer product sources of PFASs are difficult to untangle, and this study extends work identifying consumer product pathways for exposure to these chemicals and draws attention to food packaging and dental floss as modifiable sources. Although information about behavior was collected several years after the blood sample, this may not be a grave limitation because serum PFAS levels reflect long-term behavioral averages and people’s habits are likely consistent.

Among African Americans, but not other participants, eating prepared food from coated cardboard containers was associated with higher levels of four of the six PFAS chemicals evaluated (PFOA, PFNA, PFDeA, and PFOS). The differences in associations observed among African American and non-Hispanic white participants may be mediated by differences in the types of prepared food consumed most frequently: In this study, African Americans ate French fries more often than non-Hispanic whites, so we infer that they may also consume more fast food such as hamburgers, which are sold in paper wrappers. Recent data on fast food packaging show that fluorinated chemicals are detected more frequently in paper wrappers than in paperboard containers [21], so if French fries are a proxy for some exposure from food in paper wrappers, this could contribute to the differences in associations observed among African American and non-Hispanic white participants. Our questionnaire did not directly assess exposure to paper food wrappers. Harris et al. asked more generally about fast food consumption and found that eating fast food at least twice a week contributed to somewhat higher levels of PFOA, PFNA, and Me-PFOSA-AcOH in children in a model mutually adjusted for race [13].

Flossing with Oral-B Glide was associated with higher levels of PFHxS. All three Glide products that we tested contained fluorine, consistent with available information that Oral-B Glide is made with PTFE and supporting our hypothesis that Oral-B Glide is a potential exposure source for PFASs. In addition, three other flosses also tested positive for fluorine, including two of three store-brand products advertised as “compare to Oral-B Glide” on the package, and one described online as “single strand Teflon^®^ fiber” [51]. Our question did not distinguish non-flossers from people who floss with products other than Oral-B Glide, so the availability of PTFE-based flosses other than Oral-B Glide likely weakened the associations in our study, since these participants are categorized as “never” despite having a similar exposure source as the “ever” group. Our product testing results suggest that PTFE-based flosses are widely available, but that consumers can use advertising claims to help identify them. Although most of the PFAS content is expected to be polymerized in the filament, previous product testing has shown that carboxylic PFASs are detected in PTFE-based dental floss [27, 31]; no testing has been performed for the sulfonic PFASs. We were surprised to find the strongest association with PFHxS rather than the carboxylic PFASs, but proprietary production practices and formulations limit our ability to predict possible exposures. This is the first evidence that flossing with PTFE-based dental floss could contribute to an individual’s body burden of PFASs, but additional data are required to verify this finding, for example, demonstrating the potential for PFASs in floss to migrate into saliva or onto hands.

As expected, living in a city with a PFAS-contaminated water supply was associated with higher serum concentrations of PFAS chemicals. We found a significant association with three of the six PFAS chemicals evaluated (PFOA, PFNA, and PFHxS). Despite the potential for UCMR3 data to misclassify some participants—for example, those served by a small water supply not monitored under UCMR3 or by a private well, or who live in a city served by multiple public water supplies—the positive associations that we observed are consistent with findings from Hurley et al. [14] and emphasize that contaminated drinking water can be a substantial contributor to PFAS exposure. Having stain-resistant treated furniture and carpets were associated with higher levels of PFNA in all participants and PFDeA in non-Hispanic whites. Treated furniture and carpets are known sources of PFASs including PFNA and PFDeA [31], although previous studies of exposure have detected links with sulfonic, rather than carboxylic, PFASs [13, 52].

While our analysis did not identify specific behavioral pathways contributing to racial disparities in exposure, the difference in PFHxS could be mediated by factors associated with education. More education was associated with higher PFHxS among non-Hispanic whites, and a greater proportion of non-Hispanic whites had at least a Bachelor’s degree in our study. While education may partially reflect socioeconomic status, Nelson et al. found that racial differences in exposure persisted after controlling for family income [12]. More likely, there are PFAS-related behaviors not included in our model that vary by education in non-Hispanic whites. Neither education nor any other variables measured in this study were explanatory for the observed race difference in PFOA.

Consuming microwave popcorn and eating food prepared with nonstick cookware had no significant association with PFAS levels, consistent with findings from Wu et al. [35]. In contrast to previous studies, we did not detect an association between PFAS levels and consumption of seafood in the past month [17]. Although we did not account for seafood consumption at restaurants, we observed similar rates of seafood consumption in this study. However, our power may have been limited by the smaller subsample of participants asked this question.

Our study is limited by the number and location of participants, nearly all of whom lived in California. However, exposures in our study were largely comparable with those of similar-aged women in NHANES, which is designed to collect a nationally representative sample. This suggests that our smaller population—which was half African American—was typical in its PFAS exposure. Future work should include persons of Hispanic ethnicity, who often have lower exposures in NHANES analyses [9–12], and Asian Americans, whose exposures have not been characterized. Investigating the reasons for differences in exposure by race or ethnicity can help identify major exposure pathways.

A strength of this study was the opportunity to collect detailed behavioral information related to exposure from consumer products. While NHANES collects comprehensive information about diet, the few questions it includes about consumer product behaviors have well-established links to health (e.g., using sunscreen) and are not likely related to PFAS exposure. Our survey allowed consideration of a wider range of behaviors, including exploratory exposure routes, and could also target specific product types. For example, NHANES did not distinguish between different floss products when it asked about frequency of dental flossing. Our results can inform priorities for future studies that require a short questionnaire or are not designed to uncover new pathways of exposure.

Our understanding of sources of PFAS exposure remains incomplete, however. The R^2^ values for our mutually adjusted models are comparable with those obtained by Harris et al. [13], who used a similar analytical approach to assess predictors of PFAS exposure in children. Behaviors that may contribute to exposure but were not measured in this study are one source of unexplained variation, such as consuming food sold in paper wrappers. Occupational exposure to PFAS can be a major source for workers who manufacture these chemicals or frequently use PFAS-containing products, such as firefighters or ski technicians [53–55], although we expect few of our participants to have such occupational histories. Other unexplained variation can be attributed to the use of binary predictor variables, and the noise associated with self-reported behavioral data. The semi-structured interviews gave insight into the reliability of the self-report: for example, we learned that participants had difficulty identifying “nonstick cookware” but could easily check the brand of floss that they use. Because this study leveraged already-collected biomonitoring data, the behavioral predictors report on participant activity several years after the blood draws. However, with half-lives roughly between 3 and 8 years (for PFOA, PFOS, and PFHxS) [56], blood levels of PFASs are representative of long-term behavioral averages. In addition, we expect that participants’ behaviors are generally similar over time, especially at the coarse level of categorization used in this study. Changes in behavior over time would introduce some measurement error to our results, but we cannot evaluate the magnitude of the error since we did not collect information about changes in behavior over time.

While this study did not capture all the potentially important sources of PFASs, our results strengthen the evidence for exposure to PFASs from food packaging and implicate exposure from PTFE-based dental floss for the first time—a finding that warrants prompt follow-up in a future study. Environmental contamination by PFASs, for example in drinking water, remains a major public health threat, but our results also support removing PFASs from products as a way to reduce human exposure. For now, altering consumer product behaviors is one way for individuals to lower their personal exposure to these harmful chemicals.

## Supplementary information

Supplementary Information
