# Supplementary material for: Serum concentrations of PFASs and exposure-related behaviors in African American and non-Hispanic white women
Source: J Expo Sci Environ Epidemiol. 2019 Jan 8;29(2):206–17. doi: 10.1038/s41370-018-0109-y (PMC6380931; doi:10.1038/s41370-018-0109-y)
Supplement: Supplementary file 1 — Supplementary Information [file 41370_2018_109_MOESM1_ESM.docx]

**SUPPORTING INFORMATION**

**Serum concentrations of PFASs and exposure-related behaviors in African American and non-Hispanic white women**

Authors: Katherine E. Boronow,^1^* Julia Green Brody,^1^ Laurel A. Schaider,^1^ Graham F. Peaslee,^2^ Laurie Havas,^3^ Barbara A. Cohn^4^

^1^Silent Spring Institute, Newton, MA

^2^University of Notre Dame, Notre Dame, IN

^3^Child Health and Development Studies Participant Advisory Council, Berkeley, CA

^4^Child Health and Development Studies, Public Health Institute, Berkeley, CA

*Corresponding author:

320 Nevada Street, Suite 302

Newton, MA 02460

617-332-4288 ext. 228

boronow@silentspring.org

Content of Supporting Information:

9 pages (including cover sheet)

4 Tables

**PIGE method for fluorine analysis**

Approximately 2 cm of floss was cut from each package of floss with methanol washed scissors. The sample was taped over an aluminum target frame with a 1-cm hole through which a proton beam was passed. The beam of 3.4 MeV protons was produced at the Hope College Ion Beam Analysis Laboratory, and approximately 3–5 electrical nanoAmperes of beam *ex vacuo* impinged on each target for 180 seconds. Resultant gamma-rays were measured by a calibrated high-purity germanium detector (20% efficiency) located at approximately 75° to the beam. The characteristic gamma rays of 110 keV and 197 keV from the excitation of ^19^F were analyzed off-line for each spectrum, using IDL software (Harris Geospatial Solutions, Inc.) to integrate the peaks and to perform background subtraction. Using these data together with faraday cup measurements of the beam current before and after each measurement, we calculated total ^19^F counts/microCoulombs of beam on target and a statistical error. Inorganic fluorine standards were analyzed by mixing a known mass of sodium fluoride (Sigma-Aldrich) with Cellulose binder (SPEX Corp.) and pressing the homogenized mixture into a self-supporting pellet that could be used as a target in the ion beam. Measurements of commercial Teflon^®^ tape were used to compare polymeric fluorine solids (polytetrafluoroethylene or PTFE) to the inorganic fluoride standards.

Table S1. Characteristics of participants (n=178) using original questionnaire items and response levels.

| Questionnaire item  Response levels | N (%) | | |  |
| --- | --- | --- | --- | --- |
|  | Non-African American | African American | All |  |
| What is the highest level of education that you have completed? | | | |  |
| Less than high school | 2 (2) | 5 (6) | 7 (4) |  |
| High school diploma | 36 (40) | 32 (37) | 68 (35) |  |
| Associate’s degree | 4 (4) | 10 (11) | 14 (7) |  |
| Vocational-technical training | 1 (1) | 7 (8) | 8 (4) |  |
| Bachelor’s degree | 27 (30) | 26 (30) | 53 (28) |  |
| Master’s degree | 16 (18) | 5 (6) | 21 (11) |  |
| Doctoral degree | 1 (1) | 2 (2) | 3 (2) |  |
| Professional degree | 4 (4) | 0 (0) | 4 (2) |  |
| In the last month, did you eat any fish or seafood purchased at the grocery store or caught in California waters? | | | | |
| No | 23 (34) | 21 (30) | 44 (32) |  |
| Yes | 45 (66) | 48 (70) | 93 (68) |  |
| Not asked | 23 | 18 | 41 |  |
| In the last month, how often did you use Oral B Glide dental floss? | | | | |
| Never or almost never | 46 (51) | 48 (55) | 94 (49) |  |
| Several times a month | 12 (13) | 8 (9) | 20 (10) |  |
| 2 or more times a week | 14 (15) | 12 (14) | 26 (14) |  |
| Every day | 19 (21) | 19 (22) | 38 (20) |  |
| In the last month, how often did you eat food prepared using nonstick cookware? | | | | |
| Never or almost never | 20 (22) | 21 (24) | 41 (21) |  |
| Several times a month | 21 (23) | 26 (30) | 47 (24) |  |
| 2 or more times a week | 35 (38) | 28 (32) | 63 (33) |  |
| Every day | 15 (16) | 12 (14) | 27 (14) |  |
| In the last month, how often did you eat popcorn made in microwave popcorn bags? | | | | |
| Never or almost never | 74 (81) | 63 (72) | 137 (71) |  |
| Several times a month | 13 (14) | 15 (17) | 28 (15) |  |
| 2 or more times a week | 2 (2) | 8 (9) | 10 (5) |  |
| Every day | 2 (2) | 1 (1) | 3 (2) |  |
| In the last month, how often did you eat take-out French fries? | | | | |
| Never or almost never | 74 (81) | 44 (51) | 118 (61) |  |
| Several times a month | 16 (18) | 32 (37) | 48 (25) |  |
| 2 or more times a week | 1 (1) | 11 (13) | 12 (6) |  |
| Every day | 0 (0) | 0 (0) | 0 (0) |  |
| In the last month, how often did you eat pizza that came in a pizza box, either frozen from a grocery store or from a take-out restaurant? | | | | |
| Never or almost never | 53 (58) | 60 (69) | 113 (59) |  |
| Several times a month | 36 (40) | 25 (29) | 61 (32) |  |
| 2 or more times a week | 2 (2) | 1 (1) | 3 (2) |  |
| Every day | 0 (0) | 1 (1) | 1 (1) |  |
| In the last month, how often did you eat take-out food—other than pizza or French fries—from coated cardboard containers? | | | | |
| Never or almost never | 62 (68) | 47 (54) | 109 (57) |  |
| Several times a month | 26 (29) | 33 (38) | 59 (31) |  |
| 2 or more times a week | 3 (3) | 6 (7) | 9 (5) |  |
| Every day | 0 (0) | 1 (1) | 1 (1) |  |
| During the last 5 years, thinking about the furniture and carpets you have brought into your home, how many items were treated to make them stain resistant? | | | | |
| None | 57 (63) | 62 (71) | 119 (62) |  |
| One | 20 (22) | 5 (6) | 25 (13) |  |
| 2-4 | 13 (14) | 18 (21) | 31 (16) |  |
| 5 or more | 1 (1) | 2 (2) | 3 (2) |  |
| During the last 5 years, how often have you or someone in your household used a spray treatment on your furniture or carpets to make them stain resistant? | | | | |
| Never | 72 (79) | 71 (82) | 143 (74) |  |
| Once | 9 (10) | 5 (6) | 14 (7) |  |
| 2-4 times | 8 (9) | 7 (8) | 15 (8) |  |
| 5 times or more | 2 (2) | 4 (5) | 6 (3) |  |

Table S2. Spearman correlation coefficients for frequently detected PFAS analytes. Coefficients in bold are significant at p ≤ 0.001.

|  | PFOA | PFNA | PFDeA | PFHxS | PFOS | Me-PFOSA-AcOH |
| --- | --- | --- | --- | --- | --- | --- |
| PFOA | — |  |  |  |  |  |
| PFNA | **0.73** | — |  |  |  |  |
| PFDeA | **0.67** | **0.73** | — |  |  |  |
| PFHxS | **0.51** | **0.39** | **0.35** | — |  |  |
| PFOS | **0.67** | **0.65** | **0.66** | **0.56** | — |  |
| Me-PFOSA-AcOH | 0.12 | 0.08 | 0.13 | 0.10 | 0.21 | — |

Table S3. Unadjusted associations between participant behavior and serum PFAS concentration with tests for statistical interaction by race. We report the overall significance of the interaction term and the magnitude and significance of the race-specific marginal associations.

| Predictor | | | Percent change in PFAS concentration (95 percent confidence interval) | | | | | | | | | | | | | |  |
| --- | --- | --- | --- | --- | --- | --- | --- | --- | --- | --- | --- | --- | --- | --- | --- | --- | --- |
| Response levels | | PFOA | | PFNA | | PFDeA | | | PFHxS | | | PFOS | | Me-PFOSA-AcOH | | |  |
| Intercept | | 91.6 (58.7, 131.4) | | -26.5 (-37.6, -13.5) | | -76.7 (-80.9, -71.5) | | | 4.2 (-15.8, 28.9) | | | 352.4 (267.9, 456.2) | | -79.5 (-84.2, -73.5) | | |  |
| Race | |  | | |  | |  | | |  | | |  | |  | | |
| NHW | | ref | | | ref | | ref | | | ref | | | ref | | ref | | |
| AA | | -25.5 (-42.8, -3.1)* | | | 1.6 (-19.1, 27.7) | | -12.7 (-34.2, 15.7) | | | -31.4 (-49.1, -7.5)* | | | -10.8 (-33.2, 19.1) | | -5.1 (-33.9, 36.1) | | |
| Glide floss | | | | | | | | | | | | | | | |  |  |
| Never | | ref | | | ref | | ref | | | ref | | | ref | | ref | | |
| Ever*Race | | p = 0.32 | | | p = 0.33 | | p = 0.67 | | | p = 0.84 | | | p = 0.98 | | p = 0.50 | | |
| Ever (NHW) | | 18.1 (-9.7, 54.4) | | | 20 (-4.9, 51.3) | | 2.3 (-23.2, 36.3) | | | 30.8 (-3.4, 77.1) | | | 14.5 (-14.7, 53.6) | | 5.9 (-26.6, 52.8) | | |
| Ever (AA) | | -2.9 (-26.3, 27.9) | | | 1.7 (-19.9, 29.1) | | 12 (-16.6, 50.4) | | | 25 (-8.4, 70.7) | | | 15.1 (-14.9, 55.7) | | 27.2 (-12.8, 85.4) | | |
| Intercept | | 114.8 (61.4, 185.9) | | | -16.3 (-34.7, 7.4) | | -71.2 (-78.8, -60.9) | | | 23.9 (-10.6, 71.7) | | | 434.2 (290.1, 631.6) | | -79.6 (-86.2, -69.8) | | |
| Race | |  | | |  | |  | | |  | | |  | |  | | |
| NHW | | ref | | | ref | | ref | | | ref | | | ref | | ref | | |
| AA | | -43.8 (-62.3, -16.3)** | | | -17.4 (-41.6, 16.9) | | -24.1 (-50.4, 16.2) | | | -24.6 (-52.2, 18.9) | | | -16.3 (-46, 29.9) | | 0.1 (-42.1, 73.3) | | |
| Non-stick cookware | |  | | |  | |  | | |  | | |  | |  | | |
| Never | | ref | | | ref | | ref | | | ref | | | ref | | ref | | |
| Ever*Race | | p = 0.29 | | | p = 0.42 | | p = 0.36 | | | p = 0.53 | | | p = 0.77 | | p = 0.91 | | |
| Ever (NHW) | | -4 (-30.5, 32.7) | | | -5.1 (-28.4, 25.7) | | -22.6 (-45.2, 9.3) | | | -5 (-34.4, 37.4) | | | -12 (-38.3, 25.7) | | 4.4 (-33.1, 62.8) | | |
| Ever (AA) | | 22.6 (-11, 68.9) | | | 11.6 (-15.5, 47.5) | | -2.8 (-30.9, 36.8) | | | -19.7 (-44.3, 15.7) | | | -5.1 (-33.3, 35) | | 8 (-30.4, 67.7) | | |
| Intercept | | 105.7 (65.2, 156.1) | | | -24.8 (-37.9, -9.1) | | -76.7 (-81.6, -70.6) | | | 14.6 (-11.5, 48.6) | | | 325.5 (233.9, 442.3) | | -81.4 (-86.4, -74.7) | | |
| Race | |  | | |  | |  | | |  | | |  | |  | | |
| NHW | | ref | | | ref | | ref | | | ref | | | ref | | ref | | |
| AA | | -50 (-63.8, -30.8)*** | | | -16 (-36.7, 11.3) | | -27.3 (-48.6, 2.9) | | | -38.2 (-57.8, -9.3)* | | | -22.4 (-45.8, 11) | | -3.8 (-39.1, 52) | | |
| Coated cardboard containers | | | | |  | |  | | |  | | |  | |  | | |
| Never | | ref | | | ref | | ref | | | ref | | | ref | | ref | | |
| Low*Race | | p = 0.12 | | | p = 0.89 | | p = 0.47 | | | p = 0.83 | | | p = 0.76 | | p = 0.84 | | |
| Low (NHW) | | 5.8 (-19.8, 39.7) | | | 12.7 (-11.5, 43.5) | | 5.6 (-21.5, 42.2) | | | 7.6 (-22.5, 49.4) | | | 27.4 (-6.3, 73.2) | | 19.7 (-19.1, 77.1) | | |
| Low (AA) | | 46 (8.2, 96.9)* | | | 15.5 (-11, 49.8) | | 24.2 (-9.9, 71.2) | | | 13.3 (-20.5, 61.5) | | | 36.7 (-1.8, 90.4) | | 26.8 (-16.9, 93.5) | | |
| High*Race | | **p = 0.003** | | | **p = 0.025** | | **p = 0.005** | | | p = 0.39 | | | **p = 0.034** | | p = 0.85 | | |
| High (NHW) | | -28.1 (-58.6, 24.8) | | | -3.7 (-40.4, 55.5) | | -24.9 (-58.4, 35.6) | | | -7.9 (-52, 76.9) | | | -18.1 (-55.5, 50.8) | | 38.3 (-36.5, 201.1) | | |
| High (AA) | 107.8 (36.8, 215.8)*** | | | | 92.3 (33.7, 176.8)*** | | 119.3 (40.1, 243.4)*** | | | | 31.8 (-19.7, 116.3) | | 88.7 (18.8, 199.8)** | | 52.1 (-15.7, 174.5) | | |
| Intercept | | 102.1 (74.4, 134.1) | | | -22.7 (-32, -12.1) | | -77.2 (-80.6, -73.3) | | | 21.7 (2.8, 44.1) | | | 364 (294.6, 445.6) | | -78.3 (-82.3, -73.4) | | |
| Race | |  | | |  | |  | | |  | | |  | |  | | |
| NHW | | ref | | | ref | | ref | | | ref | | | ref | | ref | | |
| AA | -36.2 (-48.6, -20.7)*** | | | | -6 (-22.2, 13.6) | | -11.6 (-30, 11.6) | -39.5 (-52.9, -22.4)*** | | | | | -13.9 (-32.2, 9.3) | | 1 (-25.2, 36.4) | | |
| Microwave popcorn | |  | | |  | |  | | |  | | |  | |  | | |
| Never | | ref | | | ref | | ref | | | ref | | | ref | | ref | | |
| Ever*Race | | p = 0.48 | | | p = 0.67 | | p = 0.89 | | | p = 0.16 | | | p = 0.87 | | p = 0.73 | | |
| Ever (NHW) | | 16.8 (-16.9, 64.2) | | | 23.4 (-8.4, 66.1) | | 20.7 (-16.3, 74) | | | -11.4 (-40.1, 31) | | | 24.8 (-14.2, 81.6) | | -15 (-47, 36.1) | | |
| Ever (AA) | | 37.6 (1.6, 86.4)* | | | 13.2 (-13.1, 47.6) | | 24.9 (-9.8, 73.1) | | | 29.4 (-8.8, 83.4) | | | 30.4 (-6.7, 82.1) | | -4.8 (-37.5, 44.9) | | |
| Intercept | | 99.8 (66.4, 139.9) | | | -26.2 (-37, -13.6) | | -79.9 (-83.4, -75.6) | | | 19.7 (-2.8, 47.4) | | | 361 (277.1, 463.5) | | -78.8 (-83.5, -72.8) | | |
| Race | |  | | |  | |  | | |  | | |  | |  | | |
| NHW | | ref | | | ref | | ref | | | ref | | | ref | | ref | | |
| AA | | -27 (-43.1, -6.3)* | | | -1.3 (-20.4, 22.3) | | 9 (-16.1, 41.8) | | | -28.7 (-46.4, -5.3)* | | | -4.2 (-27.1, 26) | | -3.4 (-31.4, 35.9) | | |
| Stain-resistant carpet and furniture | | | | |  | |  | | |  | | |  | |  | | |
| None | | ref | | | ref | | ref | | | ref | | | ref | | ref | | |
| One or more*Race | | p = 0.35 | | | p = 0.6 | | **p = 0.052** | | | p = 0.37 | | | p = 0.41 | | p = 0.53 | | |
| One or more (NHW) | | 9.2 (-16.6, 42.9) | | | 20.4 (-4.6, 51.9) | | 41.3 (6.5, 87.6)* | | | -1.2 (-27.3, 34.3) | | | 11 (-17.4, 49.1) | | -1.4 (-31.8, 42.6) | | |
| One or more (AA) | | -9.7 (-32.3, 20.6) | | | 9.9 (-14.3, 41) | | -6.6 (-31.1, 26.6) | | | -19.7 (-42.2, 11.6) | | | -7.6 (-32.7, 26.9) | | 17.3 (-21, 74.2) | | |
| Intercept | | 105.1 (79.5, 134.4) | | | -20.3 (-29, -10.5) | | -76.6 (-79.7, -73) | | | 16.2 (-0.2, 35.3) | | | 374.4 (309.4, 449.8) | | -79.5 (-82.9, -75.3) | | |
| Race | |  | | |  | |  | | |  | | |  | |  | | |
| NHW | | ref | | | ref | | ref | | | ref | | | ref | | ref | | |
| AA | -33.6 (-45.2, -19.5)*** | | | | -8.9 (-22.8, 7.6) | | -11.5 (-28, 8.8) | -33.8 (-46.9, -17.6)*** | | | | | -11.3 (-28.2, 9.7) | | 4.8 (-19.6, 36.7) | | |
| City served by a PFAS-contaminated water supply | | | | | | |  | | |  | | |  | |  | | |
| No | | ref | | | ref | | ref | | | ref | | | ref | | ref | | |
| Yes*Race | | p = 0.89 | | | p = 0.46 | | p = 0.59 | | | p = 0.46 | | | p = 0.50 | | p = 0.17 | | |
| Yes (NHW) | | 90.1 (-22.8, 368.2) | | | 47.3 (-32.4, 221) | | 51.5 (-42.3, 298) | | | 197.2 (6.4, 730.2)* | | | 139.6 (-11.4, 547.7) | | 229.6 (-4.9, 1042.1) | | |
| Yes (AA) | | 105 (7.6, 290.9)* | | | 112.3 (21.5, 270.8)** | | 111 (5.7, 321.2)* | | | 83.6 (-12, 283.2) | | | 57.1 (-22.9, 220.2) | | 12.9 (-53.6, 174.8) | | |
| Intercept | | 106.4 (69.9, 150.9) | | | -17.9 (-30.7, -2.8) | | -79.6 (-83.4, -75) | | | -0.8 (-20.4, 23.7) | | | 323.1 (241.9, 423.5) | | -80.1 (-84.7, -74) | | |
| Race | |  | | |  | |  | | |  | | |  | |  | | |
| NHW | | ref | | | ref | | ref | | | ref | | | ref | | ref | | |
| AA | | -27 (-43.8, -5.2)* | | | -3.3 (-22.9, 21.3) | | 6.6 (-19.2, 40.7) | | | -18.1 (-39, 10) | | | 3.8 (-22, 38.1) | | 2.9 (-28.1, 47.2) | | |
| Education (Bachelor’s degree or more) | | | | |  | |  | | |  | | |  | |  | | |
| No | | ref | | | ref | | ref | | | ref | | | ref | | ref | | |
| Yes*Race | | p = 0.33 | | | p = 0.55 | | p = 0.14 | | | **p = 0.065** | | | p = 0.15 | | p = 0.89 | | |
| Yes (NHW) | | 1.5 (-22.4, 32.7) | | | -3.9 (-23.9, 21.4) | | 32.2 (-0.6, 75.7) | | | 41.1 (4.2, 91.1)* | | | 28.9 (-3.9, 72.8) | | 11.1 (-23.1, 60.6) | | |
| Yes (AA) | | -16.4 (-36.9, 10.9) | | | -13.2 (-32.1, 10.9) | | -3.5 (-28.5, 30.2) | | | -7 (-32.4, 28) | | | -5.6 (-30.6, 28.6) | | 15.3 (-21.7, 69.8) | | |

*p ≤ 0.05; **p ≤ 0.01; ***p ≤ 0.001

Table S4. Presence or absence of fluorine in a selection of dental flosses as determined by particle-induced γ-ray emission (PIGE) spectroscopy.

| Product name on package | N^1^ | Detectable fluorine |
| --- | --- | --- |
| Colgate Total Dental Floss Mint | 2 | Yes |
| CVS Health SuperSlip Ease Between Waxed^2^ | 2 | Yes |
| Oral-B Glide Pro-Health Mint | 2 | Yes |
| Oral-B Glide Pro-Health Original | 5 | Yes |
| Crest Glide Deep Clean Cool Mint Floss^3^ | 1 | Yes |
| Signature Care Mint Waxed Comfort Floss^2^ | 1 | Yes |
| CVS Unwaxed | 1 | -- |
| Desert Essence Tea Tree Oil Dental Tape | 1 | -- |
| EcoDent Gentle Floss Premium Dental Floss with Essential Oils Mint Vegan Waxed | 1 | -- |
| Johnson & Johnson Listerine Cool Mint Mint Floss | 1 | -- |
| Johnson & Johnson Reach Clean Paste Icy Mint Woven Floss | 1 | -- |
| Johnson & Johnson Reach Mint Waxed | 1 | -- |
| Reach Mint Waxed | 2 | -- |
| Reach Waxed Unflavored | 1 | -- |
| Oral-B Complete Deep Clean Ultra Mint | 1 | -- |
| Oral-B Satin Floss Mint | 1 | -- |
| Rite-Aid Premium Waxed Mint Floss^2^ | 1 | -- |
| Tom's of Maine Naturally Waxed Antiplaque Flat Floss | 1 | -- |

^1^Number of packages tested. Duplicate pairs from a package are listed as one sample. Results of all duplicate pairs (n = 5), and of all packages of the same product, were in agreement.

^2^Product packaging includes the phrase “Compare to Oral B Glide Pro-Health.”

^3^Crest Glide was subsequently rebranded as Oral B Glide by its parent company Procter & Gamble.
